# American Registry of Ambulatory or acutely decompensated heart failure (AMERICCAASS Registry): First 1000 patients

**DOI:** 10.1002/clc.24182

**Published:** 2023-11-30

**Authors:** Juan Esteban Gómez‐Mesa, Juliana María Gutiérrez‐Posso, Manuela Escalante‐Forero, Brayan Daniel Córdoba‐Melo, Paula Andrea Cárdenas‐Marín, Eduardo R. Perna, Mádelyn Raquel Valle‐Ramos, Germán Camilo Giraldo‐González, Noel Alberto Flórez‐Alarcón, Ida Fabiola Rodríguez‐Caballero, Cristian Núñez‐Carrizo, Luz Teresa Cabral‐Gueyraud, Sarah Raquel Marte‐Arias, Elizabeth Ashley Hardin, Amada Álvarez‐Sangabriel, María Eugenia Menjívar‐De Ramos, Kwame van der Hilst, Licurgo Jacob Cruz‐Díaz, Sergio Roberto Fausto Ovando, Luis Arturo Rodríguez, Juan Pablo Escalante, Gabriela Ormaechea‐Gorricho, Norberto Raul Bornancini, María Juliana Rodríguez‐González, Sebastián Campbell‐Quintero, Raquel E. González‐Hormostay, Guillermo Oviedo‐Pereira, Guillermo Trout‐Guardiola, Juan Justiniano Encina, Ana Margarita Jerez‐Castro, Mark Drazner, Daniel Quesada‐Chaves, Alexander Romero‐Guerra, Víctor Alejandro Rossel‐Mariángel, Mario Speranza

**Affiliations:** ^1^ Department of Cardiology Fundación Valle del Lili Cali Colombia; ^2^ Department of Health Sciences Universidad Icesi Cali Colombia; ^3^ Fundación Valle del Lili Centro de investigaciones Clínicas Cali Colombia; ^4^ Instituto De Cardiología J. F. Cabral Department of Heart Failure and Pulmonary Hypertension Corrientes Argentina; ^5^ Department of Internal Medicine Hospital Regional de Zacapa Ciudad de Zacapa Guatemala; ^6^ Department of Cardiology Todo por el Corazón cardiology center Manizales Colombia; ^7^ Instituto Nacional de Cardiología “Prof. Dr. Juan Adolfo Cattonni” Asunción Paraguay; ^8^ Clínica El Castaño Multipurpose Unit, Echocardiography and Ambulatory San Juan Argentina; ^9^ CIMAC, Multipurpose Unit, Echocardiography and Ambulatory San Juan Argentina; ^10^ Hospital Nacional de Itaugua, Department of Cardiology Itaugua Paraguay; ^11^ Department of Cardiology Clínica Universitaria Unión Médica del Norte Santiago Dominican Republic; ^12^ Department of Internal Medicine University of Texas Southwestern Medical Center Dallas Texas USA; ^13^ Emergency Department and Coronary Care Unit Instituto Nacional de Cardiología Ignacio Chávez Mexico city Mexico; ^14^ Department of Cardiology Centro de Cardiología y Ortopedia (CCO) San Salvador El Salvador; ^15^ Department of Cardiology Thorax Center Paramaribo Paramaribo Suriname; ^16^ Department of Cardiology Academic Hospital Paramaribo Paramaribo Suriname; ^17^ Department of Cardiology Hospital Regional Universitario “Presidente Estrella Ureña” Santiago Dominican Republic; ^18^ Department of Cardiology Cardiocentro Chimaltenango Chimaltenango Guatemala; ^19^ Cardiology Unit Hospital Roosevelt Guatemala city Guatemala; ^20^ Instituto Cardiovascular de Rosario, Heart Failure Transplantation and Pulmonary Hypertension Unit Rosario Argentina; ^21^ Department of Internal Medicine Hospital de Clínicas “Dr. Manuel Quintela” Montevideo Uruguay; ^22^ Department of Cardiology Centro Médico Talar Buenos Aires Argentina; ^23^ Department of Heart Failure and Transplantation LaCardio Bogota Colombia; ^24^ Department of Cardiology Clínica Medilaser Florencia Colombia; ^25^ ASCARDIO, Heart Failure Unit Barquisimeto Venezuela; ^26^ ASCARDIO, Coronary Intensive Care Unit Barquisimeto Venezuela; ^27^ ASCARDIO, Echocardiography Laboratory Barquisimeto Venezuela; ^28^ Department of Internal Medicine Instituto De Previsión Social Asuncion Paraguay; ^29^ Department of Cardiology GrupSalud Santa Marta Colombia; ^30^ Hospital Universitario Japonés, Department of Medicine Santa Cruz de la Sierra Bolivia; ^31^ Department of Cardiology Instituto de Cardiología y Cirugía Cardiovascular La Habana Cuba; ^32^ Department of Cardiology University of Texas Southwestern Medical Center Dallas Texas USA; ^33^ Department of Cardiology Hospital San Vicente de Paul Heredia Costa Rica; ^34^ Department of Cardiology Hospital Santo Tomas Panama City Panama; ^35^ Department of Cardiology Hospital del Salvador Santiago Chile; ^36^ Department of Cardiology Instituto Nacional del Tórax Santiago Chile; ^37^ Department of Cardiology Hospital Clínica Bíblica San Jose Costa Rica

**Keywords:** ambulatory, America, heart failure, hospitable

## Abstract

**Background:**

About 80% of cardiovascular diseases (including heart failure [HF]) occur in low‐income and developing countries. However, most clinical trials are conducted in developed countries.

**Hypothesis:**

The American Registry of Ambulatory or Acutely Decompensated Heart Failure (AMERICCAASS) aims to describe the sociodemographic characteristics of HF, comorbidities, clinical presentation, and pharmacological management of patients with ambulatory or acutely decompensated HF in America.

**Methodology:**

Descriptive, observational, prospective, and multicenter registry, which includes patients >18 years with HF in an outpatient or hospital setting. Collected information is stored in the REDCap electronic platform. Quantitative variables are defined according to the normality of the variable using the Shapiro–Wilk test.

**Results:**

This analysis includes data from the first 1000 patients recruited. 63.5% were men, the median age of 66 years (interquartile range 56.7–75.4), and 77.6% of the patients were older than 55 years old. The percentage of use of the four pharmacological pillars at the time of recruitment was 70.7% for beta‐blockers (BB), 77.4% for angiotensin‐converting enzyme inhibitor (ACEI)/angiotensin II receptor blocker (ARB II)/angiotensin receptor‐neprilysin inhibitor (ARNI), 56.8% for mineralocorticoid receptor antagonists (MRA), and 30.7% for sodium–glucose cotransporter type‐2 inhibitors (SGLT2i). The main cause of decompensation in hospitalized patients was HF progression (64.4%), and the predominant hemodynamic profile was wet‐warm (68.3%).

**Conclusions:**

AMERICCAASS is the first continental registry to include hospitalized or outpatient patients with HF. Regarding optimal medical therapy, approximately a quarter of the patients still need to receive BB and ACEI/ARB/ARNI, less than half do not receive MRA, and more than two‐thirds do not receive SGLT2i.

## Graphical abstract

In this first report of the AMERICCAASS registry, most patients have reduced (or mildly reduced) ejection fraction. Regarding OMT, approximately a quarter of patients are not receiving BB or ACEI/ARB/ARNI, less than half are not receiving MRA, and more than two‐thirds are not receiving SGLT2i.

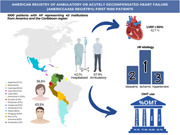

## 1 INTRODUCTION

Heart failure (HF) is a heterogeneous clinical syndrome characterized by a structural or functional heart alteration, constituting a global public health problem.^1^, ^2^ In Europe, a 1%–2% prevalence is estimated in adults, going higher as age increases, being almost greater than 10% in patients older than 70 years old.^3^ The American Heart Association estimates that in the United States, by 2030, there will be a prevalence of HF of more than 8 million people over 18 years old.^4^, ^5^

Mortality and early rehospitalization rates for HF globally are high, with poor long‐term survival, even with availability and access to optimal medical therapy (OMT). Acute decompensation of HF generates millions of hospitalizations annually,^6^ being one of the most common causes of hospital admissions, with approximately 900 000 hospital discharges per year in the United States and 81 500 in the United Kingdom.^7^ In South America, hospital readmission rates of 33.4% and 28.1% are reported at 90 days and 6 months, with in‐hospital mortality of 11.7% and overall mortality of 24.5%.^8^ HF is one of the significant causes of morbidity and mortality in the elderly, and its prognosis may be worse in Latin American countries due to social conditions, differences in etiology, disease severity, and availability and access to OMT.^8^

About 80% of cardiovascular diseases (including HF) occur in low‐income and developing countries. However, most clinical studies are carried out in developed countries, such as the United States and European countries.^9^ Given the different conditions in Latin America and the Caribbean region, It is impossible to extrapolate experiences from North American and European studies and registries based on diagnosis and treatment strategies in patients with HF. Therefore, it became necessary to implement the American Registry of Ambulatory or Acutely Decompensated Heart Failure (AMERICCAASS), which seeks to identify the intrinsic characteristics of the population of the American continent (North, Central, and South America) and the Caribbean region with HF, thus which will allow the development of strategic approaches, guide the implementation of preventive measures and appropriate treatments that will be reflected in the improvement of health care indicators in patients with HF.

## 2 METHODS

### 2.1 Design and supervision

The AMERICCAASS registry is an observational, descriptive, prospective, and multicenter study. The protocol was designed by the Board of Directors of the Inter‐American Council on Heart Failure and Pulmonary Hypertension (CIFACAH) of the Inter‐American Society of Cardiology (SIAC), which also acts as the Scientific Committee and Coordinator of the registry. The Clinical Research Center (CIC) of the Fundación Valle del Lili (FVL) in Cali, Colombia, provides technical and logistical support for its implementation. Medical institutions from the countries of the American continent and the Caribbean region (Spanish‐ and English‐speaking) are invited to participate. Data collection is completed by the principal investigator and the sub‐investigator of each participating institution through the electronic platform REDCap (Research Electronic Data Capture). This study was performed in line with the principles of the Declaration of Helsinki. Approval was granted by the Biomedical Research Ethics Committee of the Fundación Valle del Lili (November 13, 2018/No. 313‐2018)

### 2.2 Data source, data quality control, and statistical considerations

The participants are men or women, over 18 years old, with a diagnosis of HF, divided into two groups: hospitalized and outpatient. The diagnosis of HF is defined as a history of this pathology or a new HF event and receiving pharmacological management for this condition. Patients are excluded if they are hospitalized for another cardiovascular or non‐cardiovascular illness and who present decompensation of their HF during hospitalization, if they are on the active list for heart transplantation or for implantation of a ventricular assist device as destination therapy, if they are in a palliative care program for HF or another condition, if they have any inability to comply with scheduled follow‐ups or if their life expectancy is less than 6 months due to a state other than HF.

Recruitment started on April 1, 2022 and is scheduled to end on November 30, 2023. Each participating institution must include at least 60 patients. For those outpatients, a follow‐up will be carried out at 12 months, while for those hospitalized at the time of recruitment, a follow‐up will be carried out at 1 month, 6 months, and 12 months after discharge. Sociodemographic variables (age, sex, race, nationality), comorbidities, pharmacological treatment, laboratory test, and cardiovascular procedures (diagnostic and therapeutic) performed during hospitalization or follow‐up are being registered.

To avoid bias, the participating researchers from each center were trained to fill out the database correctly, taking into account the inclusion and exclusion criteria. For quality control, a random sample of 10% of the patients recruited by each institution was taken to assess the veracity of the data collected. For the statistical analysis, a univariate analysis was performed; the normality of the variables was determined using the Shapiro–Wilk test, and accordingly, they were expressed in the median and interquartile range. Categorical variables are presented as proportions, and groups are compared using Fisher's exact test. Quantitative variables were compared according to distribution using Student's *t* or non‐parametric test, in the case of independent samples, the Mann–Whitney test, and the Wilcoxon test for paired samples.

## 3 RESULTS

**Figure 1:**
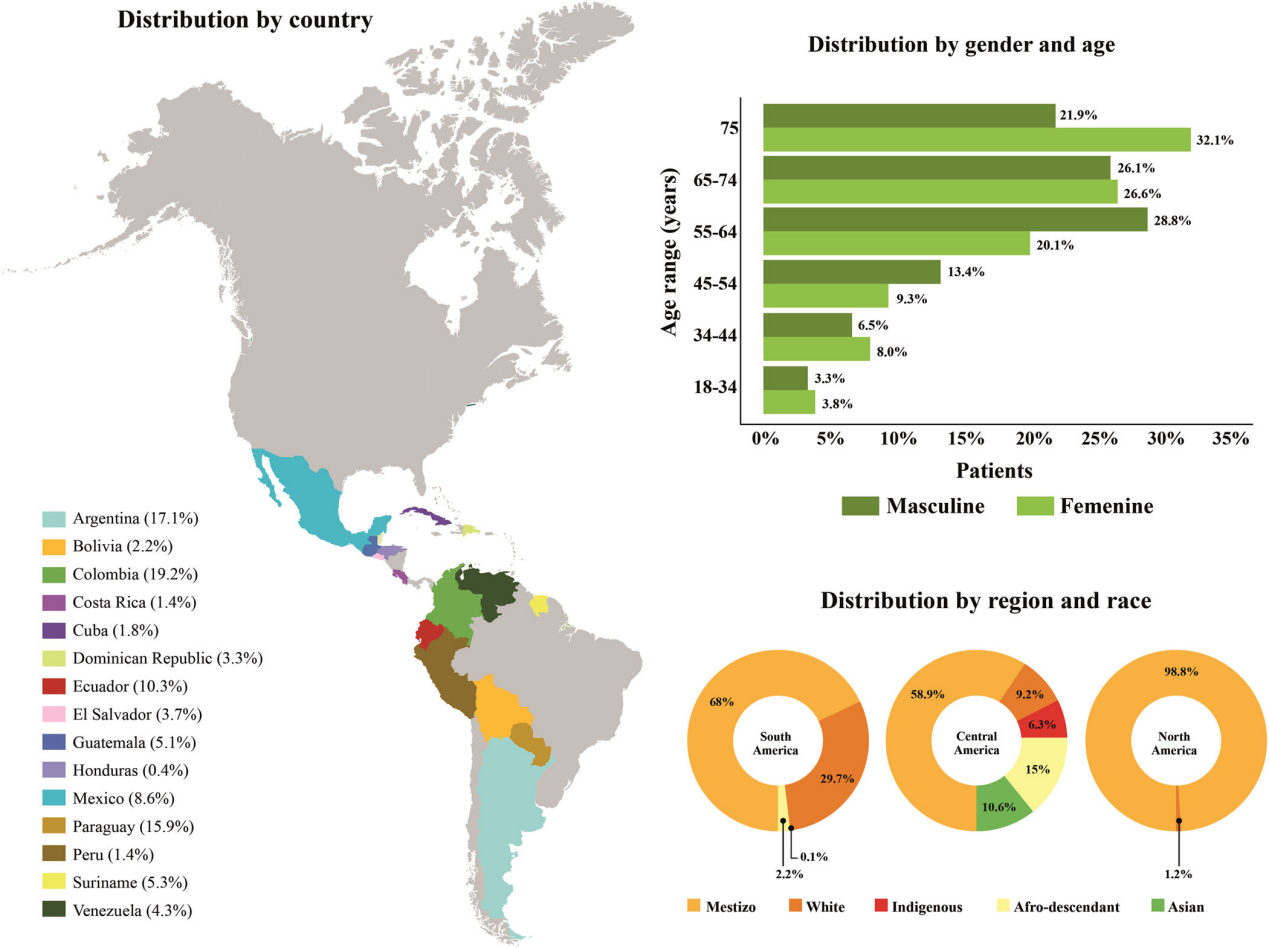
Demographic characteristics.

The first 1000 patients included in this analysis were recruited by 43 institutions from 15 countries of the American continent. 41.9% of the hospitals were public, 48.8% private, and 9.3% belonged to both regimes, with more than half being academic institutions (62.8%). 63.5% of the patients were men, with a median age of 66 years (interquartile range [IQR] 56.7–75.4), 77.6% of the patients were older than 55 years old. The most prevalent race was mestizo (68.8%) (Figure 1). Of these first 1000 recruited patients, 57.9% were ambulatory and 42.1% were hospitalized, 66.8% had a history of HF, and 77.7% had been previously hospitalized. The main comorbidities were arterial hypertension (65.3%), dyslipidemia (39.7%), coronary disease (33.7%), atrial fibrillation (28.3%), and diabetes mellitus (28%). The main HF etiologies were ischemic (37.1%), idiopathic (14.5%), and hypertensive (14.4%). About lifestyle, the use of tobacco stands out (34.1%), 85.9% consumed it previously, and 14.1% consumed it at the time of the recruitment. Alcohol intake was reported by 27.7% of patients, with the majority reporting casual or social consumption (46.2%) (Table 1).

**Table 1:** Demographics of the population.

Characteristic	Patients *n* (%) *N* = 1000
Age, years	66 (56.7, 75.4)^a^
Sex (male/female)	63.5/36.5
Comorbidities	
Arterial hypertension	653 (65.3)
Dyslipidemia	397 (39.7)
Coronary heart disease	337 (33.7)
Atrial fibrillation/atrial flutter	283 (28.3)
Diabetes mellitus	280 (28)
Previous COVID infection	251 (25.1)
Severe valvular regurgitation	211 (21.1)
Renal disease	170 (17)
Lifestyle	
Smoking	341 (34.1)
Previous	293 (85.9)
Current	48 (14.1)
Alcohol consumption	277 (27.7)
Once or twice per week	112 (40.4)
3–5 times per week	24 (8.7)
5–7 times per week	13 (4.7)
Occasional‐social	128 (46.2)

Abbreviation: IQR, interquartile range.

^a^ Median (IQR).

Regarding HF characteristics in hospitalized and ambulatory patients, reduced left ventricular ejection fraction (LVEF) was the most frequent finding (49.3% and 59.5%, respectively). The predominant symptom in both groups was dyspnea on exertion (73.1%) and the main sign was edema in the lower limbs (45.3%). Most patients were overweight (38.6% of hospitalized patients and 41% of outpatients). Regarding blood pressure, hospitalized patients had a median systolic and diastolic blood pressure of 120.0 mmHg (106.0–140.0) and 75.0 mmHg (66.0–90.0), respectively, while the values for outpatients were 120.0 mmHg (RIC 109.0–133.2) and 70.0 mmHg (RIC 63.0–80.0), respectively. Inpatients had higher creatinine (1.2 mg/dL [IQR 1.0–1.5]) and blood urea nitrogen (32.0 mg/dL [IQR 19.6–51.3]) than outpatients (1.1 mg/dL [IQR 0.9–1.4] and 26.0 mg/dL [IQR 18.0–46.0], respectively) (Table 2).

**Table 2:** Baseline clinical features, physical examination, and LVEF.

Clinical variables	Hospitalized *N* = 421, *n* (%)	Ambulatory *N* = 579, *n* (%)
NYHA		
I	11 (2.7)	120 (21.2)
II	67 (16.1)	318 (56.3)
III	212 (51.1)	108 (19.1)
IV	125 (30.1)	19 (3.4)
Signs		
Edema in lower limbs	293 (69.6)	160 (27.6)
Peripheral edema	280 (66.5)	129 (22.3)
Crepitus	271 (64.4)	83 (14.3)
External jugular venous pressure (>6 cm)	211 (50.1)	74 (12.8)
Hepatomegaly	150 (35.6)	39 (6.7)
Pleural effusion	135 (32.1)	17 (2.9)
Symptoms		
Dyspnea on exertion	346 (82.2)	385 (66.5)
Dyspnea at rest	289 (68.6)	55 (9.5)
Palpitations/tachycardia	154 (36.6)	77 (13.3)
Bloating/abdominal pain	139 (33.0)	31 (5.4)
LVEF		
40–50	59 (14.0)	95 (21.3)
<40	207 (49.3)	266 (59.5)
>50	154 (36.7)	86 (19.2)
Weight	75.0 (65.0, 85.8)^a^	74.0 (65.0, 84.0)^a^
Size	165.0 (158.0, 170.0)^a^	165.0 (158.0, 170.0)^a^
BMI		
Normal (BMI <25)	119 (30.6%)	163 (33.1)
Overweight (BMI 25–29.9)	150 (38.6%)	202 (41.0)
Obesity (BMI 30–>40)	120 (30.8)	128 (26.0)
No data	32	86
Systolic blood pressure	120.0 (106.0, 140.0)^a^	120.0 (109.0, 133.2)^a^
Diastolic blood pressure	75.0 (66.0, 90.0)^a^	70.0 (63.0, 80.0)^a^
Heart rate	85.0 (72.0, 101.0)^a^	70.0 (64.0, 80.0)^a^
Creatinine	1.2 (1.0, 1.5)^a^	1.1 (0.9, 1.4)^a^
Blood urea nitrogen	32.0 (19.6, 51.3)^a^	26.0 (18.0, 46.0)^a^

Abbreviations: BMI, body mass index; IQR, interquartile range; LVEF, left ventricular ejection fraction; NYHA, New York Heart Association.

^a^ Median (IQR).

Most hospitalized patients were admitted from the emergency room (72.7%), with a median hospital stay of 7.0 days (IQR 4.0–13.0). Their main cause of decompensation was HF progression (44.2%), followed by atrial fibrillation (12.1%) and acute coronary syndrome (9%). The predominant hemodynamic profile on admission was “wet‐warm” (68.3%), and the main clinical presentation was decompensated HF (64.4%). (Table 3).

**Table 3:** Information on hospitalized patients.

Clinical variable	Hospitalized *N* = 421, *n* (%)
Main causes of decompensation	
Heart failure (progression)	186 (44.2)
Atrial fibrillation	51 (12.1)
Acute coronary syndrome	38 (9.0)
Pharmacological nonadherence	33 (7.8)
Infection	28 (6.7)
Arterial hypertension	23 (5.5)
Hemodynamic profile	
Wet, warm	285 (68.3)
Wet, cold	61 (14.6)
Dry, warm	60 (14.4)
Dry, cold	11 (2.6)
No data	4
Clinical presentation	
Decompensated heart failure	261 (64.4)
Pulmonary edema	51 (12.6)
Cardiogenic shock	31 (7.7)
Heart failure/acute coronary syndrome	25 (6.2)
Hypertensive heart failure	21 (5.2)
Right heart failure	9 (2.2)
Admission to ICU	131 (31.1)
Days in ICU (days)	6.0 (3.0, 11.0)^a^
Days in non‐ICU (days)	4.0 (2.5, 8.0)^a^
Length of hospital stay (days)	7.0 (4.0, 13.0)^a^

Abbreviations: ICU, intensive care unit; IQR, interquartile range.

^a^ Median (IQR).

Regarding OMT use at recruitment, beta‐blockers (BBs) were used by 70.7% of patients, carvedilol being the most widely used (48.9%), mineralocorticoid receptor antagonists (MRA) were used by 56.8% of patients, and sodium‐glucose cotransporter type 2 inhibitors (SGLT2i) by 30.7%. Moreover, 30.4% of the patients used angiotensin II receptor blockers (ARB II), 25.2% angiotensin–neprilysin receptor inhibitors (ARNI), and 21.8% angiotensin‐converting enzyme (ACE inhibitors). Finally, only 217 patients (21.7%) received OMT, defined by BBs, MRA, SGLT2i, and ARB II, ARNI, or ACE inhibitors, regardless of the dose of each therapy. The most frequently prescribed diuretic was furosemide (88.4%) (Table 4).

**Table 4:** Pharmacological treatment.

Pharmacological treatment	Patients (*n*, %) *N* = 1000
ARB II	304 (30.4)
Losartan	177 (58.2)
Valsartan	65 (21.4)
Candesartan	14 (4.6)
Other	48 (15.8)
Sacubitril/valsartan	252 (25.2)
ACEI	218 (21.8)
Enalapril	191 (87.6)
Lisinopril	9 (4.1)
Ramipril	8 (3.7)
Captopril	7 (3.2)
Other	3 (1.4)
Beta‐blockers	707 (70.7)
Carvedilol	346 (48.9)
Bisoprolol	221 (31.3)
Metoprolol succinate	75 (10.6)
Nebivolol	53 (7.5)
Other	12 (1.7)
Aldosterone antagonist	568 (56.8)
Spironolactone	497 (87.5)
Eplerenone	71 (12.5)
SGLT2 inhibitors	307 (30.7)
Dapagliflozin	201 (65.5)
Empagliflozin	106 (34.5)
Diuretics	605 (60.5)
Furosemide	535 (88.4)
Thiazide	41 (6.8)
Others	29 (4.8)

Abbreviations: ACEI, angiotensin‐converting enzyme inhibitors; ARB II, angiotensin II receptor blockers; SGLT2, sodium–glucose cotransporter type 2.

## 4 DISCUSSION

This study analyzed the first 1000 patients recruited in the AMERICCAASS registry by 43 institutions from 15 countries of the American continent to characterize their sociodemographic aspects, comorbidities, clinical presentation, and use of OMT.

A systematic review and meta‐analysis of HF in Latin America, where 143 studies were included, reports a mean age of 60.34 ± 8.98 years and a mean LVEF of 36 ± 9%,^10^ while our study reports a median age of 66 years old and an LVEF of 35% (IQR 27.0–46.0). The most frequently reported comorbidities were arterial hypertension (62%), anemia (40%), chronic kidney disease (25%), and atrial fibrillation (22%),^10^ similar to what was found in our report, where 65.3% they had arterial hypertension, 17% had chronic kidney disease and 28.3% atrial fibrillation.

In 2019, Luciana Gioli‐Pereira et al. described a cohort of patients with HF in Brazil (GENIUS‐HF). A predominance of hypertensive (26.0%), ischemic (21.9%), and chagasic (17.0%) etiology was observed.^11^ In our sample, ischemic (23.7%) and hypertensive (14.4%) etiology also predominated. Chagas disease, on the other hand, represents only 3.9%, which could be explained due to the high prevalence of Chagas disease in Brazil,^12^ and at the moment of this report, Brazil is not participating in the registry.

Cardiovascular risk factors, mainly smoking and obesity, are rising in developing countries. A cardiovascular risk assessment study carried out in Latin America, which included six countries (Argentina, Brazil, Colombia, Chile, Guatemala, and Mexico) found that abdominal obesity (48.5%) was the main risk factor, followed by dyslipidemia (40.8%) and smoking (38.4%)^13^; which correlates with the prevalence of these two cardiovascular risk factors in our study, where 39.7% of the patients had dyslipidemia and 34.1% of the patients had a history of smoking.

On the other hand, obesity in the Latin American and Caribbean region represents an important health problem related to diet and the recent and rapid demographic transition that leads to a sedentary lifestyle and poor eating habits.^14^ It was found in this study that 35.2% of the patients were overweight (BMI 25.0–29.9 kg/m^2^), while 24.8% were obese (BMI >30 kg/m^2^). Although this prevalence varies according to the cohort, the percentage of overweight patients coincides with that described in the literature. However, the rate of obesity is much lower, which could be related to most of the large cohorts being in North America, where obesity levels exceed those of the general population.^15^, ^16^

HF is associated with a high burden of cardiac and noncardiac complications, even higher in patients with diabetes mellitus.^17^ This comorbidity has a prevalence that varies in different registries; in our study, it was found to be 28%, which is close to that reported by large multinational studies, such as the “Global Study of Congestive Heart Failure” (G‐CHF), which reports a prevalence of diabetes mellitus of 31% in the population with HF^18^ and the “International Registry to Evaluate Medical Practice with Longitudinal Observation for the Treatment of Heart Failure” (REPORT‐HF), which described a prevalence of 31% in Central and South America.^19^ Although renal failure, described as serum creatinine greater than 1.5 mg/dL, is frequently reported in hospitalized patients and is found in more than half of patients hospitalized for acute HF,^20^ in our study, we reported a lower median serum creatinine of 1.2 mg/dL (1.0–1.5) in hospitalized patients and 1.1 mg/dL (0.9–1.4) in outpatients, being not a common complication during hospitalization in our population.

Of the 23 047 patients recruited by the G‐CHF registry until 2020, 2849 patients from South America (12.4%) were included; of these, 405 (14.2%) were inpatients and 2440 (85.6%) outpatients.^18^ In our population, we found a higher percentage of hospitalized patients, 421 (42.1%). Regarding vital signs, Héctor Gonzáles‐Pacheco et al. described in a retrospective study including 7759 patients with acute HF hospitalized in a Latin American university hospital, that the median systolic blood pressure was 130.0 mmHg (104.0–139.0) and a median heart rate of 90.0 bpm (75.0–104.0).^21^ The median for these two vital signs was lower in our population, with a median systolic blood pressure of 120.0 mmHg (106.0–140.0) and a heart rate of 85.0 bpm (72.0–101.0).

In the vast majority of studies, the worsening or progression of HF is the main cause of hospital admission, representing up to 70%–80% in some studies.^17^ The North American OPTIMIZE‐HF registry and the Eurasian registry of the GREAT network have investigated precipitating conditions in patients with HF, finding acute coronary syndromes, arrhythmias (mainly atrial fibrillation), and infections to be the most frequent causes.^22^ Of the identifiable factors in our study, the leading cause of decompensation was found to be worsening or progression of HF (44.2%), followed by atrial fibrillation (12.1%) and acute coronary syndrome (9%).

The ESC‐HF‐LT registry collected hospitalization data from 6629 patients with HF. Regarding their clinical presentation at the time of admission, 61.1% presented as decompensated HF, 14.4% as acute coronary syndrome, 13.2% as pulmonary edema, 4.8% as hypertensive HF, 3.5% as right HF, and 2.9% as cardiogenic shock.^23^ These results are similar to our analysis, where the predominant clinical phenotype was decompensated HF. This same registry classified the patients hospitalized for HF based on the clinical signs of congestion/hypoperfusion in the profiles “warm‐dry” (9.9%), “wet‐warm” (69.9%), “wet‐cold” (19.8%), and “dry‐cold” (0.4%).^24^ The distribution of these four clinical profiles was similar in our registry, with most patients classified as “wet‐warm” and a small proportion as “dry‐cold.”

A study derived from the ASCEND‐HF registry in 2014 included 5094 patients admitted to 224 hospitals. Of these, 1944 (38.2%) were admitted to ICU. This registry included 344 patients from Latin America, of whom 76 (22%) were admitted to ICU with a median ICU stay of 3.0 days (2.0–6.0) and a median total stay of 8.0 (5.0, 12.0) days for this group; and for the group not admitted to ICU, the median total hospital stay was 7.0 (4.0, 10.0) days.^25^ In our study, we reported a longer ICU stay of 6.0 days (3.0, 11.0), although the total in‐hospital stay was similar.

As recommended by the 2021 European Society of Cardiology Guidelines for Managing HF, the use of an ACEI/ARB II or ARNI, a BB, a MRA, and a SGLT2i, is defined as the OMT or the four fundamental pillars for the management of HF.^3^ This registry found that regarding OMT, 77.4% of the patients received ACEI/ARB II or ARNI, 70.7% received beta blockers, 56.8% received MRA, and 30.7% received SGLT2i. Of those patients, only 21.7% received those four fundamental pillars of the management of HF.

## 5 LIMITATIONS

The findings of this study may be limited because it is still in its recruitment phase, and there is not at least one medical institution that represents each country in the American continent. Another limitation is that with the obtained information for this analysis, we cannot calculate survival, cardiovascular/non‐cardiovascular mortality, and rehospitalization rates,

Besides that, this report includes an important number of patients, and the information presented is valuable compared to the scarce information available about the sociodemographic, etiology, and pharmacological treatment of the American population with HF.

## 6 CONCLUSIONS

AMERICCAASS is the first continental prospective registry to include hospitalized or outpatient patients with HF that includes most of the countries of the region. At this point, the main comorbidity and etiology in our region are similar to those reported in registries from other parts of the world. Most patients have reduced (or mildly reduced) ejection fraction. Regarding OMT, approximately a quarter of the patients still need to receive BB and ACEI/ARB or ARNI, less than half do not receive MRA, and more than two thirds do not receive SGLT2i. Subsequent analyses will make it possible to identify characteristics of American patients, including clinical, imaging, and data related to the OMT recommended in the international guidelines for the diagnosis and management of HF.

## AUTHOR CONTRIBUTIONS

All authors made substantial contributions to the conception or design of the work, and the acquisition, analysis, and interpretation of data. All authors drafted the work, revising it critically for important intellectual content, approved the version to be published, and agreed to be accountable for all aspects of the work.

## CONFLICT OF INTEREST STATEMENT

The authors declare no conflict of interest.

## Data Availability

The authors confirm that the data supporting the findings of this study are available within the article or its supplementary materials.
